# Free Open Access Medical Education (FOAM) in emergency medicine: use and perceived clinical relevance from a large cross-sectional survey

**DOI:** 10.1186/s12245-026-01281-3

**Published:** 2026-07-03

**Authors:** Martin Fandler, Thorben Doll, Johannes Pott, Wiebke Turner, Philipp Gotthardt, Jan Ehlers, Julia Nitsche

**Affiliations:** 1 Chair of Didactics and Educational Research in Healthcare, Witten/Herdecke University, Witten, Germany; 2 ADAC HEMS Academy, Wessling, Germany; 3 St. Bernward Krankenhaus, Hildesheim, Germany; 4 Vinzenzkrankenhaus Hannover, Hannover, Germany; 5 Notfallguru, Winnenden, Germany

**Keywords:** Emergency medicine, Prehospital emergency medicine, Free Open Access Medical Education (FOAM), Medical education, Digital learning, Open educational resources, Knowledge translation, Continuing medical education

## Abstract

**Background:**

Free Open Access Medical Education (FOAM) refers to freely accessible digital educational resources that are increasingly being used for training, continuing education and professional development, particularly in emergency medicine. While FOAM is well established internationally, there are limited data on its use, motives and perceived relevance in German-speaking countries, with multiprofessional perspectives being underrepresented both regionally and internationally.

**Methods:**

The aim of this study was to record usage patterns, sociodemographic characteristics and subjective perceptions of the benefits, limitations and didactic significance of FOAM in German-speaking countries, with a focus on emergency medicine. An exploratory, descriptive cross-sectional study was conducted using a predominantly standardised online survey, supplemented by several open-ended questions. The survey targeted adults who were working in emergency and acute medicine in German-speaking countries or who were undergoing relevant training or studying medicine. The present evaluation was purely descriptive.

**Results:**

A total of 1,910 evaluable questionnaires were analysed. This constitutes one of the largest published datasets to date on the use of FOAM. Recruitment was conducted primarily via FOAM-related platforms and social media. Among the participants, 1,731 (90.6%) stated that they currently use FOAM. The majority of FOAM users were between 31 and 40 years old; 51% were physicians and approximately 40% were paramedics or nurses. FOAM was predominantly used regularly and was considered by the vast majority to be understandable, effective and helpful for training and continuing professional development. A high proportion of users reported that, in their own estimation, FOAM content influenced or changed their clinical decisions and was perceived to improve patient care. Moreover, approximately half of those surveyed were critical of the variable quality of the content and the lack of traditional peer review processes. Only a minority made use of interactivity in the sense of active exchange within the FOAM community.

**Conclusion:**

FOAM represents a relevant and established resource for education, training and continuing professional development in this cohort of the emergency medical community in German-speaking countries. Its high subjective relevance underscores the importance of freely accessible digital educational offerings, while interpretation beyond this cohort should be made with caution. Future research should focus in particular on quality standards, content sustainability and the objectifiable effects of FOAM on clinical outcomes.

**Supplementary Information:**

The online version contains supplementary material available at 10.1186/s12245-026-01281-3.

## Background

Free Open Access Medical Education (FOAM) refers to a globally established approach to medical education that uses freely accessible digital resources such as blogs, podcasts, videos and social media to disseminate knowledge, particularly in emergency and acute care settings [1–4].

Since its introduction in 2012, FOAM has become an increasingly relevant component of medical education, training and continuing professional development [5, 6]. Medical education research and individual didactic training are also increasingly addressing this topic [7, 8].

Key advantages of FOAM include its global, free and immediate accessibility, as well as its broad range of topics and potential for interaction. It facilitates rapid knowledge translation into clinical practice and supports self-directed, flexible learning adapted to modern learning habits. In particular, its asynchronous availability enables “just-in-time” learning, which is especially beneficial for shift-based healthcare professionals [1, 9–17].

At the same time, FOAM is discussed controversially, particularly with regard to the quality and reliability of content, the lack of traditional peer review, and the sustainability of largely volunteer-driven production, funding and the risk “eminence-based” medicine [18–27].

To date, there has been a lack of systematic data on the use, motives and perceived relevance of FOAM in emergency and acute medicine in German-speaking countries. While several international surveys—primarily from North America and the United Kingdom—have described patterns of FOAM use, these studies often focus on specific professional groups (e.g. physicians or trainees) and healthcare systems that differ substantially from the German-speaking context [5, 28, 29]. In particular, differences in training structures, the organisation of emergency care and the integration of multiprofessional teams limit the transferability of these findings.

More broadly, existing international data suggest that FOAM is predominantly used by younger healthcare professionals, trainees and students, although results remain partly inconsistent and indicate lower usage in certain occupational groups, such as emergency medical services [5, 6, 28, 30–34]. In addition, standardised approaches to measuring the reach and impact of FOAM, such as the Social Media Index, have not yet been widely established [35].

Against this background, the present study aims to provide a comprehensive, multiprofessional overview of FOAM use in German-speaking emergency and acute care settings. By including different professional groups (e.g. physicians, paramedics and nurses), this study seeks to extend existing international findings and to provide insights into a healthcare context and workforce structure that have so far been underrepresented in FOAM research.

## Methods

The aim of this study was to provide a descriptive overview of the use of Free Open Access Medical Education (FOAM) in German-speaking countries, with a focus on emergency and acute medicine. The study collected data on the sociodemographic characteristics of the respondents, usage patterns, and subjective perceptions of the advantages, disadvantages, and criticisms of FOAM and its perceived relevance for clinical practice. In addition, the study aimed to describe the prevalence and acceptance of FOAM as a freely accessible online resource for continuing education in the context of emergency medicine.

A particular focus was placed on the didactic perspective: the aim was to identify the potential that FOAM offers from the users’ point of view for medical education, training and continuing professional development, and to identify the perceived challenges in terms of content quality, application in clinical practice and the sustainability of production.

In summary, this study addresses the questions of who uses FOAM, to what extent and for what purposes, and how its benefits and limitations are assessed from the perspective of users.

This study was designed as an exploratory, descriptive cross-sectional study. The aim was to systematically record and describe the use, perceptions, advantages and disadvantages of FOAM in German-speaking countries, but not to investigate causal relationships. Owing to the objective and the heterogeneous target group, a standardised online survey was chosen as the survey method. Recruitment followed an open, non-probability sampling approach. The evaluation was purely descriptive. This approach was chosen to provide a transparent overview of the dataset, to minimise the risk of overinterpretation in an exploratory dataset, and to avoid premature hypothesis testing.

No inferential statistical analyses or predefined thresholds for statistical significance or effect size were applied, as the objective of this manuscript was descriptive rather than hypothesis-testing. Inferential statistical analyses, including subgroup comparisons, are planned as part of subsequent hypothesis-driven analyses and are therefore not reported in this manuscript.

Ethical approval was obtained from the Ethics Committee of Witten/Herdecke University, Germany (approval number: S-273/2022).

Starting in August 2022, the authors developed a questionnaire for use in an online survey. The content was based on aspects of FOAM use described in the literature as well as frequently discussed advantages, limitations and points of criticism. The questionnaire was developed based on literature and expert consensus and was internally pretested for clarity, usability and technical functionality prior to fielding. No formal validation study was performed.

After the declaration of consent and confirmation of legal age, the questionnaire focused on dividing respondents into three groups: people who use FOAM; people who do not use FOAM; and people who had used FOAM in the past but no longer do so. With respect to FOAM use, various advantages, disadvantages and points of criticism described in the literature were surveyed via a six-point Likert scale, with the extreme ends labelled “strongly disagree” and “strongly agree”. The Likert items were evaluated ordinally.

The basic questionnaire, which was completed by all groups, consisted of four questions on sociodemographic data and three thematic blocks with several items on a Likert scale: continuing education (7 items), self-efficacy and readiness to use technology (7 items) and handling digital systems (8 items). The latter was based on the ICT-SC25g, a validated instrument for assessing skills in the field of information and communication technology, which was adapted for this survey [27].

For current FOAM users, five general questions about usage and specific questions about FOAM usage were added to the basic questionnaire. These included a question on the type and extent of use (16 items on a Likert scale), a question on positive aspects of FOAM (12 items on a Likert scale) and a question on critical aspects of FOAM (13 items on a Likert scale). In addition, there were optional free text fields in which individual wishes, experiences and comments on FOAM could be entered.

In addition, the use of two of the most popular German-speaking FOAM formats “Nerdfallmedizin” and “Pin-Up Docs” was surveyed. If a respective use was indicated, a separate question with four items on a Likert scale was asked for each of these formats, relating to perceived quality, relevance and usability.

For those who had used FOAM in the past but no longer did, five general questions about previous use and one question about the use of FOAM (16 items on a Likert scale) were added to the basic questionnaire. In addition, a question was asked about the reasons for no longer actively using FOAM (13 items on a Likert scale) and optional free text fields were provided for individual experiences and reasons for discontinued use.

Careful completion of the entire questionnaire in the “former FOAM users” group took about 10–15 min, provided that no lengthy free-text responses were entered.

For those who did not use FOAM, one question was added to the basic questionnaire about the reasons for not using FOAM (13 items on a Likert scale). Here, too, there was the option to add optional free text comments.

Completing the questionnaire took approximately 20–30 min for the group of FOAM users, approximately 10–15 min for the group of former FOAM users, and approximately 5–10 min for the group of non-FOAM users, provided that no extensive free text entries were made.

The survey was conducted in German and was translated into English for this publication. Participants were excluded if they did not give their consent, did not provide information on their age, or provided clearly contradictory or implausible information.

The sample size was determined pragmatically on the basis of the open recruitment strategy and the defined survey period. Owing to the exploratory nature of the study and the lack of reliable assumptions regarding the effect size, a formal power analysis was deliberately not performed.

The primary target group was defined as German-speaking medical professionals and individuals currently undergoing training or studying in this field who work in acute and emergency medicine. The campaign was aimed particularly at physicians, nurses, paramedics, students and trainees. No restrictions were set with regard to the scope of activity in acute and emergency medicine or age, apart from the requirement of being of legal age.

The survey period ran from April 10th 2023 to June 30th 2023. A predefined survey period was used instead of a fixed sample size, given the open recruitment strategy.

The survey was promoted online via various newsletters and social media, particularly via FOAM platforms. Specifically, the survey was shared on the FOAM platforms “Nerdfallmedizin” and “Pin-Up-Docs”, which at the time of the study represented two of the largest FOAM platforms in the German-speaking region (approximately 8,000–10,000 website visits per day and around 100,000 followers across social media channels). In addition, the survey was disseminated via multiple social media channels (including YouTube, WhatsApp, Telegram, Instagram and LinkedIn), and users were encouraged to further distribute the survey within their personal networks and messaging groups. Due to this multi-channel dissemination strategy, the total number of individuals exposed to the survey invitation cannot be reliably determined, and a precise response rate could therefore not be calculated.

In order to reach people who are less digitally savvy, physical posters advertising the survey were also displayed in emergency medical services and clinics in Germany, Austria and Switzerland.

However, due to the predominantly digital recruitment process, the reported prevalence of FOAM use cannot be considered representative of the entire emergency medical workforce.

After accessing the online questionnaire, the participants first received information about the study’s objective, voluntary participation and data protection. Only after actively agreeing to the informed consent form could the questionnaire be completed.

Technical measures were used in the survey software to reduce multiple participation, in particular by restricting multiple participation via identical browser sessions. Data cleansing was carried out after the survey period had ended. Questionnaires were considered usable if they contained complete sociodemographic information and if at least the group assignment had been answered. Questionnaires without content information or that were abandoned immediately after the sociodemographic questions were excluded.

The survey was conducted via an online questionnaire provided by SoSci Survey GmbH (Munich, Germany), which offers a free survey service for academic research. The statistical analysis of this study was purely descriptive and was carried out using Microsoft Excel (Microsoft Corporation, Redmond, Washington, USA). Frequencies and percentages are reported; no inferential statistical analyses were performed.

The survey was conducted anonymously; no directly or indirectly identifiable personal data were stored. IP addresses were not collected or stored. The data were processed in accordance with the applicable data protection regulations, particularly the General Data Protection Regulation (GDPR). Participation was voluntary and could be discontinued at any time without giving reasons.

The reporting of this web-based survey was guided by the CHERRIES (Checklist for Reporting Results of Internet E-Surveys) recommendations where applicable (Supplementary) [36].

## Results

The online questionnaire was started a total of 2,430 times during the survey period. Of these, 1,910 questionnaires (78.6%) with usable answers were included in the evaluation. Questionnaires without content or those that were abandoned immediately after the sociodemographic data were collected were excluded.

The first responses were collected on 10 April 2023, and the last response was collected on 27 June 2023.

Among the 1,910 participants evaluated, 1,731 (90.6%) stated that they currently use FOAM. Thirty-five individuals (1.8%) reported having used FOAM in the past but no longer did so. One hundred and forty-four participants (7.5%) stated that they do not use FOAM.

### Characteristics of FOAM users

**Fig. 1:**
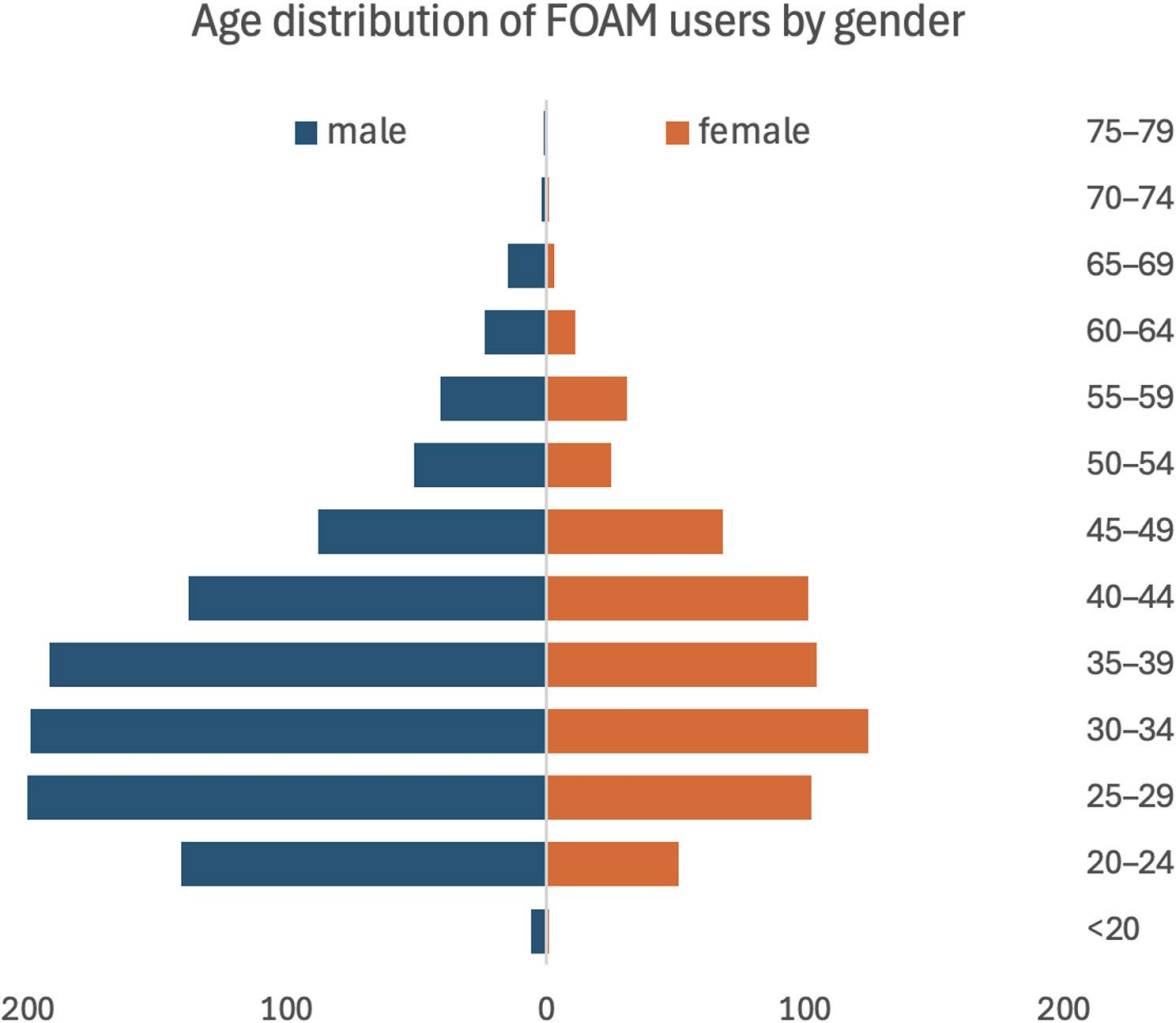
Age distribution of FOAM users by gender (FOAM-users identifying as non-binary/diverse (*n* = 6) are not shown in the figure due to small numbers.)

The largest group of FOAM users was in the 31–40 age category (550; 31.8%). Only a few participants were under 21 (22; 1.3%) or over 69 (3; 0.2%). One participant did not provide their age. The average age of FOAM users was 36.5 years (range 18–75). In terms of gender, 622 participants (35.9%) identified as female, 1103 (63.7%) as male and 6 (0.3%) as nonbinary or diverse (Table 1; Fig. 1). The age and gender distributions of FOAM users largely reflected those of the overall sample.

**Table 1:** FOAM users

**Age** (***n* = 1,730)**	36.5 ± 10.5 years (mean ± SD; range 18–75)
Gender (*n* = 1,731)	
Male	1103 (63.7%)
Female	622 (35.9%)
Nonbinary / diverse	6 (0.3%)
Occupational groups (*n* = 1 731)	
Physician	882 (51%)
Emergency medical personnel	532 (30.7%)
Nurse	143 (8.3%)
Students / Trainees	87 (5%)
Other academic degree in the health sector (excl. physicians)	68 (3.9%)
Other	19 (1.1%)

The FOAM users were predominantly physicians (882; 51%) and emergency medical personnel (532; 30.7%). Nurses accounted for 143 participants (8.3%), students and trainees for 87 (5.0%) and other academic health professionals for 68 (3.9%). Other professional groups accounted for 1.1% of FOAM users.

### Use of continuing education and FOAM

The majority of respondents stated that they spent a total of 6–10 h per month on continuing education (656; 37.9%), followed by 1–5 h (506; 29.2%) and 11–20 h (377; 21.8%). Only a few participants reported spending less than one hour or more than 40 h per month on continuing education.

**Fig. 2:**
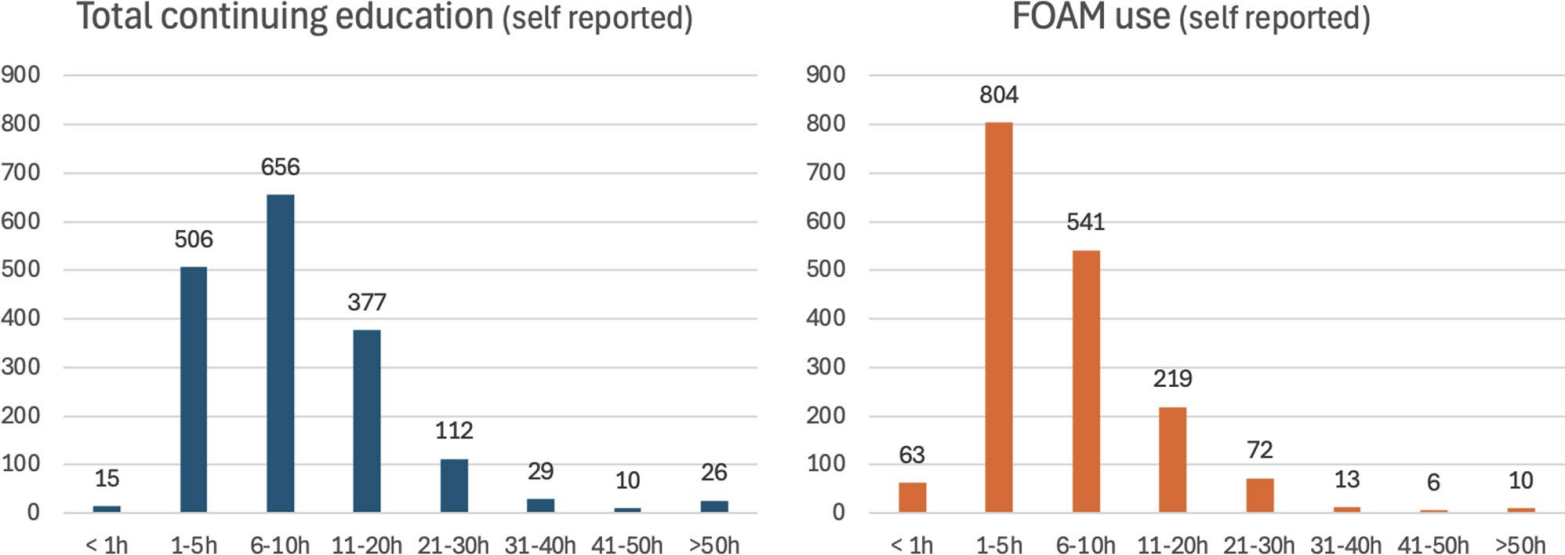
Distribution of self-reported total continuing education and FOAM use per month. Total continuing education and FOAM use were assessed as separate self-reported variables and do not represent hierarchical components

FOAM was predominantly used for 1–5 h per month (804; 46.4%) or 6–10 h per month (541; 31.3%). For 653 respondents (37.7%), FOAM use was credited as part of formal continuing education certification (e.g. Continuing Medical Education (CME) points), whereas 1,076 participants (62.2%) did not report any formal credit (Fig. 2).

### Assessment of FOAM

The vast majority of FOAM users rated FOAM positively overall. A total of 97.9% stated that they enjoyed learning with FOAM (“somewhat agree” 152; 8.8%, “agree” 592; 34.2%, “strongly agree” 952; 55%) and 99% rated the content as mostly understandable (“somewhat agree” 181; 10.5%, “agree” 803; 46.4%, “strongly agree” 728; 42.1%). 92.5% of users stated that they would like to invest more time in using FOAM if they had the time, and 99.1% would recommend its use to others.

Only a minority interact: just 20% see themselves as part of the FOAM community, and only slightly more (30.9%) exchange ideas with other users.

**Fig. 3:**
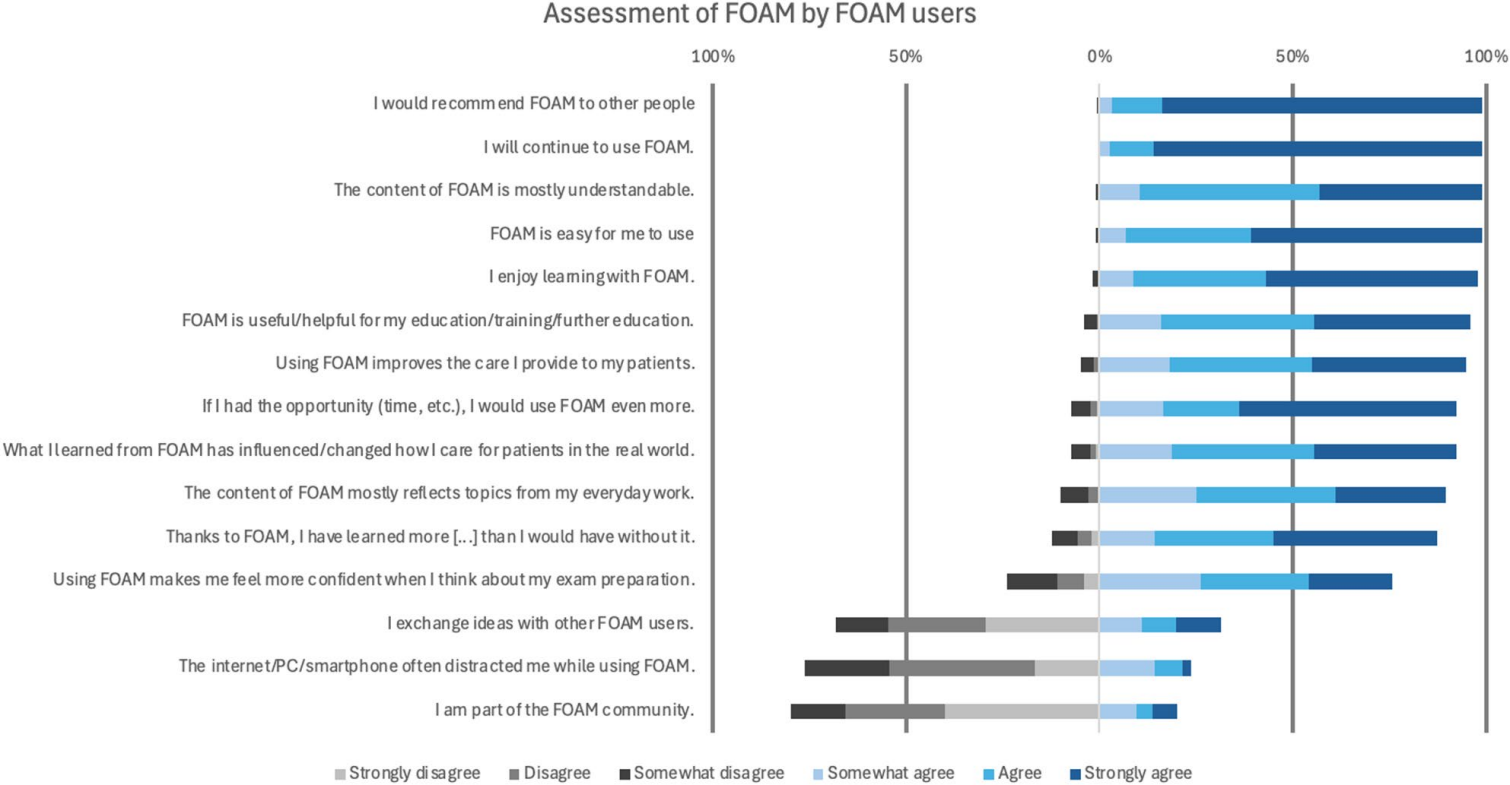
Assessment of FOAM by FOAM users

A large majority of respondents also reported that, in their own estimation, FOAM content had influenced or changed their clinical decisions (92.2%) and that this had subjectively resulted in an improvement in patient care (94.8%) (Fig. 3; Supplementary Table 1).

**Fig. 4:**
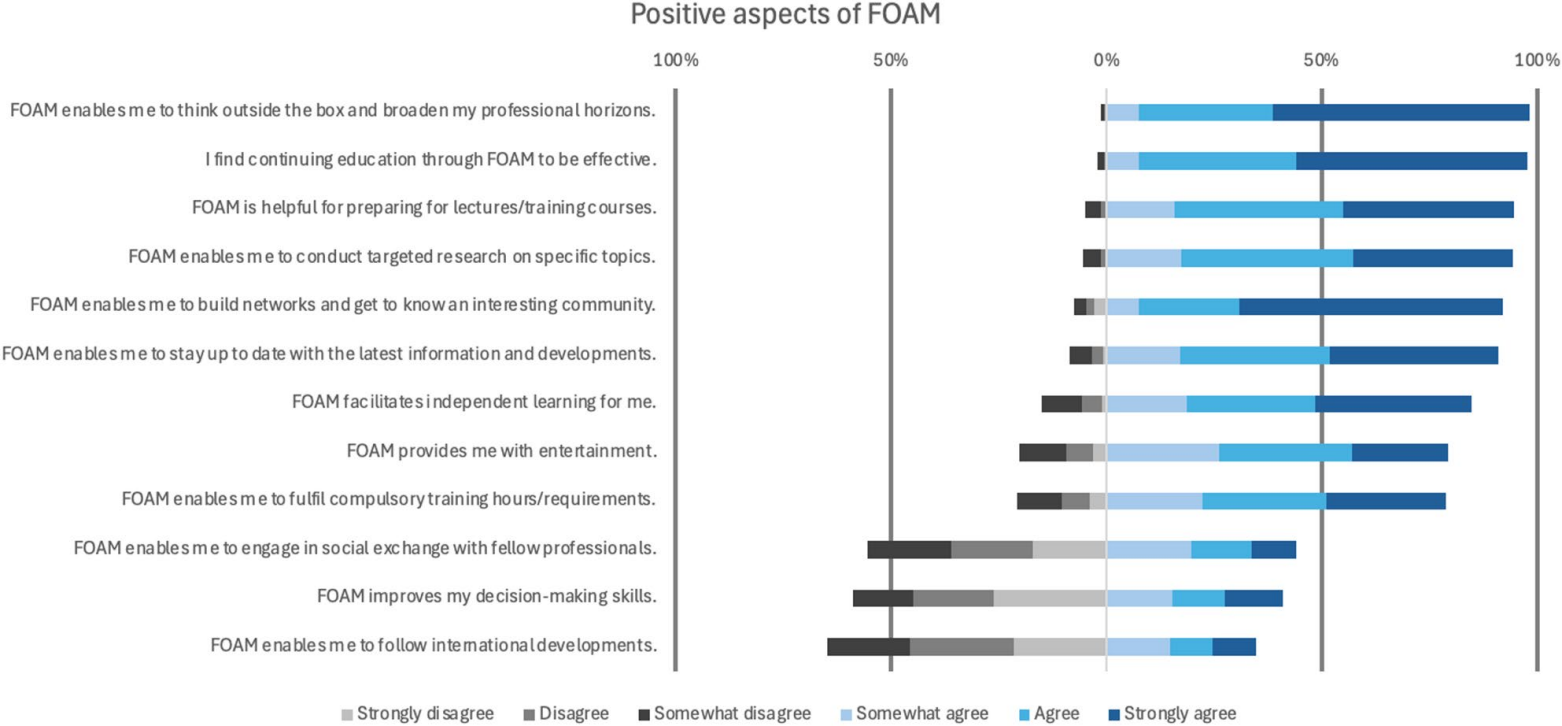
Positive aspects of FOAM

The expansion of professional horizons (98.4% agreement), the effectiveness of training (97.8%) and the support for independent learning (84.8%) were cited as particularly positive aspects of FOAM. In addition, 94.7% of users rated FOAM as helpful in preparing for training courses or lectures (Fig. 4, Supplementary Table 2). The assessment was more cautious with regard to improving one’s own decision-making and action-taking skills (41.2% agreement).

**Fig. 5:**
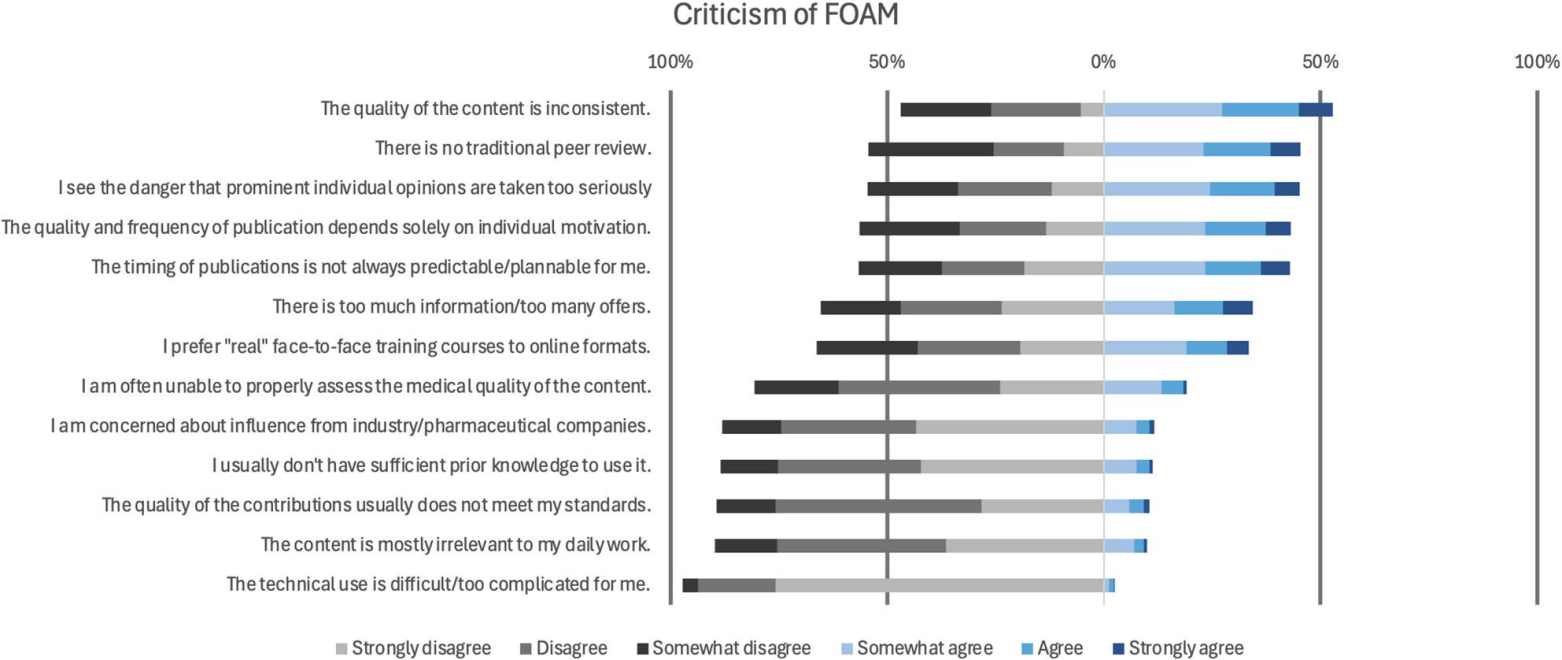
Criticism of FOAM

The variable quality of content was particularly criticised, with 52.8% of FOAM users agreeing with this assessment. The lack of traditional peer review procedures and the potential overvaluation of prominent individual opinions were considered problematic by approximately half of the respondents. The technical difficulties in using FOAM, on the other hand, played only a minor role, with 2.6% agreeing (Fig. 5, Supplementary Table 3).

## Discussion

To the authors’ knowledge, this study is one of the largest surveys published to date on the use of FOAM and the only such study in the German-speaking world [6, 29, 31]. The results indicate a high level of acceptance and regular use of FOAM, particularly among medical and emergency service personnel. The inclusion of multiple professional groups represents a particular strength, as previous studies have often focused on single professions. These findings are broadly consistent with international studies from North America and the United Kingdom, which report high levels of FOAM use, especially among younger clinicians and trainees [5, 28, 30–33].

FOAM was rated positively by the majority of users, particularly with regard to comprehensibility, perceived quality and recommendation to colleagues. This may reflect both the increasing integration of digital learning formats into routine professional development and the characteristics of the study sample, which is likely to be more receptive to FOAM due to the recruitment strategy.

The high reported influence of FOAM on clinical decision-making and patient care is notable. However, these findings should be interpreted with caution, as they are based exclusively on subjective self-assessments and may be affected by recall bias, social desirability bias and individual attitudes towards FOAM. Consequently, no conclusions can be drawn regarding objective effects on clinical outcomes. Similar limitations of self-reported educational impact have been described in other contexts, underlining the need for objective outcome measures in future research.

Concerns regarding the quality and reliability of FOAM content were expressed by approximately half of respondents, which is consistent with international literature describing an ambivalent perception of FOAM quality [19, 21, 37–39]. At the same time, errors are also reported in traditionally peer-reviewed literature [40], highlighting that content quality remains a broader challenge in medical education. Strategies such as curated content, expert review initiatives (e.g. AIR), quality checklists and community-based evaluation may support users in identifying high-quality resources [23, 28, 33, 41, 42].

Despite the interactive potential of FOAM, the present findings suggest that it is predominantly used in a receptive manner. This aligns with previous observations that passive consumption remains the dominant mode of engagement in digital medical education [43, 44]. Active interaction and international exchange appear to play a comparatively minor role.

The distribution of users across professional groups and age categories is consistent with previous studies showing higher uptake among younger healthcare professionals and trainees [5, 28, 30–33, 45]. Structural factors, such as continuing education requirements for physicians and differences in training systems across professions, may further influence usage patterns [3, 8].

The creation and maintenance of FOAM content require substantial time and resources, often without institutional support. This raises questions regarding sustainability, independence and long-term development of FOAM initiatives [46].

Despite the large sample size and broad participation of various professional groups, this study is subject to several limitations that must be taken into account when interpreting the results.

The survey was conducted in German and therefore primarily targeted participants from German-speaking countries (Germany, Austria, Switzerland). Owing to differences in healthcare systems and varying structures for education, training and continuing professional development, the results can only be applied to other countries to a limited extent.

Data collection was conducted primarily via digital channels, including social media and newsletters, particularly FOAM-related platforms. This results in a relevant selection bias in favour of FOAM-affiliated individuals, as healthcare professionals with little affinity for digital training formats are likely to be underrepresented despite additional recruitment via physical posters. The results therefore primarily reflect the perspective of individuals who are fundamentally open to digital educational offerings.

Some authors are actively involved in FOAM initiatives as non-commercial content contributors. Although these roles were unrelated to the conduct of this study and did not involve financial interests, the possibility of intellectual bias cannot be entirely excluded. To minimise potential influence, the study design, survey instrument, and descriptive analytical approach were defined prior to data analysis, and the results were reported transparently without selective emphasis.

The questionnaire used was developed specifically for this study and has not been formally validated. Although the content was based on existing publications and established concepts, the lack of formal validation limits comparability with other studies and the interpretation of individual scales. In addition, the study was designed as an exploratory, descriptive cross-sectional study without inferential statistical analyses, and the findings are based on subjective self-assessments. These factors limit comparability with other studies and preclude conclusions regarding causal relationships or objective clinical outcomes.

The sample showed a clear overrepresentation of male participants. This imbalance could limit the generalisability of the results and reflects known structural inequalities that are also found in other areas of academic medicine and the emergency medical community [40].

## Conclusions

Within this likely FOAM-affine cohort, FOAM was regularly used and predominantly rated positively by emergency and acute care professionals in German-speaking countries. The results indicate a high level of acceptance of freely accessible digital educational offerings across various professional groups within the study sample.

The respondents consider the use of FOAM to be relevant to their own education, training and continuing professional development. A majority of users stated that, in their opinion, FOAM content had influenced or changed their clinical decisions and had subjectively contributed to an improvement in patient care.

Against the backdrop of an internationally reported decline in FOAM publications in recent years [9, 47], the available data illustrate the continued active use and high perceived clinical relevance of German-language FOAM resources within this sample. The results can thus serve as a basis for systematically recording the landscape of German-language FOAM offerings and describing their further development.

Future research should focus in particular on defining and establishing quality standards, on issues related to the sustainability of FOAM projects, and on investigating objectifiable effects on clinical practice and patient care.

## Supplementary Information

Below is the link to the electronic supplementary material.

Supplementary Material 1

## Data Availability

The dataset generated during the current study is available from the corresponding author upon reasonable request and is stored in anonymised form in accordance with institutional and data protection regulations. As further predefined analyses of the dataset are ongoing, public deposition in an open-access repository is planned following completion of these analyses.

## Abbreviations

CME: Continuing Medical Education
FOAM: Free Open Access Medical Education
GDPR: General Data Protection Regulation
