# Supplementary material for: Free Open Access Medical Education (FOAM) in emergency medicine: use and perceived clinical relevance from a large cross-sectional survey
Source: Int J Emerg Med. 2026 Jul 3;19:187. doi: 10.1186/s12245-026-01281-3 (PMC13411813; doi:10.1186/s12245-026-01281-3)
Supplement: Supplementary file 1 — Supplementary Material 1 [file 12245_2026_1281_MOESM1_ESM.docx]

# Free Open Access Medical Education (FOAM) in Emergency Medicine: Use and Perceived Clinical Relevance from a Large Cross-Sectional Survey

Supplement

*Supplementary Table 1: Assessment of FOAM by FOAM users*

|  | 1 (strongly disagree) | 2 (disagree) | 3 (I tend to disagree) | 4 (somewhat agree) | 5 (agree) | 6 (strongly agree) | No reply |
| --- | --- | --- | --- | --- | --- | --- | --- |
| I would recommend FOAM to other people | 0 | 5 | 6 | 56 | 225 | 1435 | 4 |
| I will continue to use FOAM. | 0 | 0 | 8 | 44 | 198 | 1476 | 9 |
| The content of FOAM is mostly understandable. | 0 | 1 | 15 | 181 | 803 | 728 | 3 |
| FOAM is easy for me to use | 1 | 2 | 13 | 116 | 561 | 1035 | 3 |
| I enjoy learning with FOAM. | 1 | 6 | 25 | 152 | 592 | 952 | 3 |
| FOAM is useful/helpful for my education/training/further education. | 3 | 2 | 18 | 117 | 537 | 1051 | 3 |
| Using FOAM makes me feel more confident when I think about my exam preparation. | 68 | 123 | 225 | 451 | 488 | 372 | 4 |
| If I had the opportunity (time, etc.), I would use FOAM even more. | 14 | 28 | 84 | 285 | 340 | 976 | 4 |
| What I learned from FOAM has influenced/changed how I care for patients in the real world. | 15 | 27 | 87 | 326 | 634 | 637 | 5 |
| The content of FOAM mostly reflects topics from my everyday work. | 5 | 43 | 129 | 432 | 627 | 492 | 3 |
| Thanks to FOAM, I have learned more and developed my skills to a greater extent than I would have without it. | 34 | 66 | 115 | 248 | 530 | 735 | 3 |
| Using FOAM improves the care I provide to my patients. | 9 | 16 | 60 | 313 | 641 | 688 | 4 |
| I exchange ideas with other FOAM users. | 512 | 432 | 238 | 192 | 151 | 203 | 3 |
| The internet/PC/smartphone often distracted me while using FOAM. | 292 | 648 | 379 | 248 | 125 | 36 | 3 |
| I am part of the FOAM community. | 690 | 446 | 246 | 164 | 73 | 109 | 3 |

*Supplementary Table 2: Positive aspects of FOAM*

|  | 1 (strongly disagree) | 2 (disagree) | 3 (I tend to disagree) | 4 (somewhat agree) | 5 (agree) | 6 (strongly agree) | No reply |
| --- | --- | --- | --- | --- | --- | --- | --- |
| FOAM enables me to engage in social exchange with fellow professionals. | 294 | 331 | 337 | 342 | 241 | 181 | 5 |
| I find continuing education through FOAM to be effective. | 0 | 8 | 28 | 129 | 633 | 929 | 4 |
| FOAM is helpful for preparing for lectures/training courses. | 3 | 20 | 64 | 272 | 681 | 687 | 4 |
| FOAM enables me to conduct targeted research on specific topics. | 3 | 18 | 74 | 300 | 692 | 640 | 4 |
| FOAM enables me to build networks and get to know an interesting community. | 49 | 31 | 51 | 132 | 401 | 1062 | 5 |
| FOAM enables me to stay up to date with the latest information and developments. | 13 | 46 | 91 | 295 | 601 | 679 | 6 |
| FOAM facilitates independent learning for me. | 20 | 79 | 160 | 321 | 519 | 628 | 4 |
| FOAM provides me with entertainment. | 54 | 107 | 191 | 454 | 535 | 385 | 5 |
| FOAM enables me to fulfil compulsory training hours/requirements. | 66 | 113 | 182 | 386 | 496 | 483 | 5 |
| FOAM enables me to think outside the box and broaden my professional horizons. | 2 | 8 | 14 | 131 | 537 | 1035 | 4 |
| FOAM improves my decision-making skills. | 453 | 323 | 242 | 266 | 211 | 232 | 4 |
| FOAM enables me to follow international developments. | 372 | 419 | 331 | 258 | 170 | 174 | 7 |

*Supplementary Table 3: Criticism of FOAM*

|  | 1 (strongly disagree) | 2 (disagree) | 3 (I tend to disagree) | 4 (somewhat agree) | 5 (agree) | 6 (strongly agree) | No reply |
| --- | --- | --- | --- | --- | --- | --- | --- |
| The quality of the content is inconsistent. | 92 | 357 | 363 | 472 | 307 | 135 | 5 |
| There is no traditional peer review. | 161 | 278 | 501 | 400 | 265 | 120 | 6 |
| I see the danger that prominent individual opinions are taken too seriously ("eminence instead of evidence"). | 208 | 373 | 363 | 424 | 258 | 100 | 5 |
| The quality and frequency of publication depends solely on individual motivation. | 230 | 346 | 401 | 404 | 244 | 99 | 7 |
| The timing of publications is not always predictable/plannable for me. | 317 | 331 | 331 | 404 | 223 | 118 | 7 |
| There is too much information/too many offers. | 407 | 403 | 321 | 281 | 195 | 119 | 5 |
| I prefer "real" face-to-face training courses to online formats. | 333 | 410 | 403 | 330 | 162 | 87 | 6 |
| I am often unable to properly assess the medical quality of the content. | 415 | 643 | 338 | 230 | 87 | 13 | 5 |
| I am concerned about influence from industry/pharmaceutical companies. | 751 | 538 | 234 | 130 | 54 | 19 | 5 |
| I usually don't have sufficient prior knowledge to use it. | 731 | 570 | 228 | 130 | 54 | 13 | 5 |
| The quality of the contributions usually does not meet my standards. | 489 | 821 | 235 | 103 | 56 | 22 | 5 |
| The content is mostly irrelevant to my daily work. | 629 | 676 | 248 | 120 | 40 | 13 | 5 |
| The technical use is difficult/too complicated for me. | 1311 | 311 | 59 | 22 | 17 | 6 | 5 |

*Checklist for Reporting Results of Internet E-Surveys (CHERRIES)*

| **Checklist Item** | **Explanation** |
| --- | --- |
| Describe survey design | Cross-sectional web-based survey among FOAM users; convenience sample via open distribution channels. |
| IRB approval | Ethics approval was obtained; Ethics Committee of Witten/Herdecke University, Germany (approval number: S-273/2022). |
| Informed consent | Participants received study information at survey start, including purpose, voluntary participation, and data handling. Proceeding was only possible after declaration of consent and legal age. |
| Data protection | The survey was conducted anonymously; no directly or indirectly identifiable personal data were stored. IP addresses were not collected or stored. The data were processed in accordance with the applicable data protection regulations, particularly the General Data Protection Regulation (GDPR). |
| Development and testing | The content of the questionnaire was based on aspects of FOAM use described in the literature as well as frequently discussed advantages, limitations and points of criticism. The questionnaire was developed based on literature and expert consensus and was internally pretested for clarity, usability and technical functionality prior to fielding. No formal validation study was performed. Relevant parts of the basic questionnaire were based on the ICT-SC25g, a validated instrument for assessing skills in the field of information and communication technology, which was adapted for this survey. |
| Open survey versus closed survey | Open survey (accessible via public distribution; no login required). |
| Contact mode | Initial contact was made online. |
| Advertising the survey | The survey was promoted online via various newsletters and social media, particularly via FOAM websites. In addition, the survey was disseminated via multiple social media channels (including YouTube, WhatsApp, Telegram, Instagram and LinkedIn), and users were encouraged to further distribute the survey within their personal networks and messaging groups. Due to this multi-channel dissemination strategy, the total number of individuals exposed to the survey invitation cannot be reliably determined, and a precise response rate could therefore not be calculated.  In order to reach people who are less digitally savvy, physical posters advertising the survey were also displayed in emergency medical services and clinics in Germany, Austria and Switzerland. |
| Web/E-mail | Web-based survey (hosted online; automatic data capture). |
| Context | Specifically, the survey was shared on the FOAM platforms “Nerdfallmedizin” and “Pin-Up-Docs”, which at the time of the study represented two of the largest FOAM platforms in the German-speaking region (approximately 8,000–10,000 website visits per day and around 100,000 followers across social media channels).  While user-distribution was encouraged and also physical posters advertising the survey, due to the predominantly digital recruitment process, the reported prevalence of FOAM use cannot be considered representative of the entire emergency medical workforce. |
| Mandatory/voluntary | Voluntary participation. |
| Incentives | No incentives offered. |
| Time/Date | April 10th 2023 to June 30th 2023 |
| Randomization of items or questionnaires | No randomization of items. |
| Adaptive questioning | After completing the basic questionnaire, depending on the answer of “FOAM-usage” three different sets of questions were presented:  For current FOAM users, five general questions about usage and specific questions about FOAM usage were added to the basic questionnaire. These included a question on the type and extent of use (16 items on a Likert scale), a question on positive aspects of FOAM (12 items on a Likert scale) and a question on critical aspects of FOAM (13 items on a Likert scale). In addition, there were optional free text fields in which individual wishes, experiences and comments on FOAM could be entered.  In addition, the use of two of the most popular German-speaking FOAM formats "Nerdfallmedizin" and "Pin-Up Docs" was surveyed. If a respective use was indicated, a separate question with four items on a Likert scale was asked for each of these formats, relating to perceived quality, relevance and usability.  For those who had used FOAM in the past but no longer did, five general questions about previous use and one question about the use of FOAM (16 items on a Likert scale) were added to the basic questionnaire. In addition, a question was asked about the reasons for no longer actively using FOAM (13 items on a Likert scale) and optional free text fields were provided for individual experiences and reasons for discontinued use.  Careful completion of the entire questionnaire in the "former FOAM users" group took about 10-15 minutes, provided that no lengthy free-text responses were entered.  For those who did not use FOAM, one question was added to the basic questionnaire about the reasons for not using FOAM (13 items on a Likert scale). Here, too, there was the option to add optional free text comments. |
| Number of Items | The number of items varied depending on survey path (non-users, former users, current users), ranging from 32 to 75 items. |
| Number of screens (pages) | Variable.  Current FOAM-users: 3-5  Former FOAM-users: 3  Non FOAM-users: 2 |
| Completeness check | No enforced completion; the only mandatory questions was on FOAM-usage (determining the further questions sets). |
| Review step | A “Back-Button” was available until the end of the survey. |
| Unique site visitor | Not calculable due to open distribution and unknown denominator. |
| View rate (Ratio of unique survey visitors/unique site visitors) | Not applicable. |
| Participation rate (Ratio of unique visitors who agreed to participate/unique first survey page visitors) | Not applicable. |
| Completion rate (Ratio of users who finished the survey/users who agreed to participate) | 78,6% (1910/2430) |
| Cookies used | No cookies were used. |
| IP check | No IP check was used. |
| Log file analysis | None. |
| Registration | Not applicable. |
| Handling of incomplete questionnaires | Questionnaires without content or those that were abandoned immediately after the sociodemographic data were collected were excluded.  All other questionnaires were analyzed. |
| Questionnaires submitted with an atypical timestamp | Not used / applicable. |
| Statistical correction | Not used. |
